# COMMUNITY-ENGAGED STRATEGIES FOR RECRUITMENT OF ASIAN AMERICAN OLDER ADULTS

**DOI:** 10.1093/geroni/igac059.241

**Published:** 2022-12-20

**Authors:** Lan Ðoàn, Simona Kwon, Chau Trinh-Shevrin, Stella Yi

**Affiliations:** NYU Grossman School of Medicine, New York, New York, United States; NYU School of Medicine, New York, New York, United States; NYU Grossman School of Medicine, New York, New York, United States; NYU Grossman School of Medicine, New York, New York, United States

## Abstract

Asian Americans one of the fastest growing older adult populations in the U.S., and are emerging as a high-need, lower-income population. Inclusion of Asian Americans in research is critical given anti-Asian rhetoric and hate crimes targeting Asian American older adults and because Asian Americans are the fastest growing racial/ethnic group in the U.S. We focus on recruitment strategies used during the implementation of 11 primary collection data efforts, including national and regional community health resources and needs assessment surveys, launched during the COVID-19 pandemic (starting May 2020). Unique recruitment challenges included the heterogeneity of language, culture, and sociodemographic characteristics of participants, digital literacy, and survey fatigue. Effective recruitment facilitators included: prioritizing community engagement at all research stages; aligning the research purpose with community priorities; recruitment through community-based organizations and bilingual community health workers; translating survey instruments; and regularly scheduled meetings with community-based organizations to discuss the survey progress.

